# Incidence of acute pulmonary embolism, related comorbidities and survival; analysis of a Swedish national cohort

**DOI:** 10.1186/s12872-017-0587-1

**Published:** 2017-06-14

**Authors:** Therese Andersson, Stefan Söderberg

**Affiliations:** Department of Public Health and Clinical Medicine, Medicine, Umea University, -90187 Umeå, SE Sweden

**Keywords:** Pulmonary embolism, Incidence, Comorbidities, Prognosis, Sweden

## Abstract

**Background:**

The aim of the study was to determine the incidence of acute pulmonary embolism (PE) in Sweden and any regional differences. To assess short- and long-term survival analysis after an episode of PE, before and after excluding patients with known malignancies, and to determine the most common comorbidities prior to the PE event.

**Methods:**

All in-hospital patients, including children, diagnosed with acute PE in 2005 were retrieved from the Swedish National Patient Registry (NPR) and incidence rates were calculated. All registered comorbidities from 1998 until the index events were collected and survival up to 4 years after the event were calculated and compared to matched controls.

**Results:**

There were 5793 patients of all ages diagnosed with acute PE in 2005 resulting in a national incidence of 0.6/1000/year. The mean age was 70 years and 52% were women. The most frequent comorbidities were cardiac-, vascular-, infectious- and gastrointestinal diseases, injuries and malignancies. The mortality rates were more than doubled in patients with recent PE compared to that in a matched control group (49.1% vs 21.9%), and the excess mortality remained after exclusion of deaths occurring within one year and after exclusion of patients with any malignancy prior to the event.

**Conclusions:**

PE is associated with high age as well as with multiple comorbidities, and with an increased short- and long-term mortality. This study highlights the importance of a proper follow-up after an acute PE.

**Electronic supplementary material:**

The online version of this article (doi:10.1186/s12872-017-0587-1) contains supplementary material, which is available to authorized users.

## Background

Acute pulmonary embolism (PE) is associated with difficulties in respect to diagnosis, treatment and follow-up. The clinical course is highly variable ranging from asymptomatic to massive embolism with hemodynamic instability, right heart failure and death [1, 2].

PE is associated with serious co-morbidities such as malignancies, ischaemic heart disease and complicated by recurrences and development of post-thrombotic-syndrome [3–7]. In some cases, the thrombotic process initiates a chronic obstructive process of the pulmonary vessels leading to pulmonary hypertension, e.g. chronic thromboembolic pulmonary hypertension (CTEPH), a disease associated with high morbidity and mortality [8].

Despite being a frequent diagnosis, the incidence of acute PE is still uncertain. Previous studies have reported an annual incidence between 0.2 to 0.8 /1000 where the discrepancy may relate to differences in inclusion criteria [9–12]. The PE incidence in Sweden is 0.8/1000/year according to one single-centre study [13]. There are no data on regional differences in incidence rates or any north-south gradient in the country.

Today’s knowledge about concomitant diseases related to PE is mostly based on clinical trials and these patients are often not a reflection of the PE patients in real life. Recently, it was reported that venous thromboembolism and pulmonary embolism are often associated with multiple concomitant diseases [14], not least cardiovascular disease, in addition to other well-known associated conditions such as malignancies, recent trauma or surgery and previous venous thromboembolism [15–19]

Despite improving diagnostic and treatment regimes, the prognosis after an acute PE is still poor. However, the mortality in PE differs between studies mainly due to differences in the inclusion criteria, such as inclusion of PE only or a combination of any kind of venous thromboembolic disease, inclusion of hemodynamic stable patients or all patients regardless of hemodynamic state, and inclusion of autopsy verified pulmonary embolism or not. Estimates of the short-term mortality rate have varied between 17 to 48% at 30 days [20, 21] and between 19 to 30% for 6 to 12 months [3, 22–25]. Studies on long-term prognosis are few, but have indicated that mortality is increased up to 8 years after the acute event [3, 20, 25, 26]. Whether malignancies are responsible for the increased mortality in the long-term setting is unclear [22, 27].

In this study, the Swedish National Inpatient Register (IPR) was used to determine the incidence of acute PE in Sweden and its regional distribution, short- and long-term prognosis and comorbidities with the focus concomitant CVD.

## Methods

### Subjects

In Sweden, all in-patient care and concomitant data are registered in the Swedish National Inpatient Register (IPR) hosted by the Swedish National Board of Health and Welfare. This register was inaugurated in 1984 and has a national coverage of over 99%, as reporting is mandatory by law, and the validity of IPR has proven to be high [28].

The Swedish modification of ICD (*International classification of diseases)*-10, ICD-10-SE was used to identify patients with an acute PE as main or subsidiary diagnose during 2005. Altogether 5793 unique persons with a first (during 2005) PE were identified.

A control population was created by the Swedish agency of Statistics, and each unique person with PE was matched with a control person on the basis of age (± 1 day), gender and residency. This was successful in all except 5 cases (very low or very high age, few inhabitants, or incorrect registration). The combined cohort of patients with PE and their matched controls were followed until the 23th of April 2010, and 4107 deaths occurred. Altogether 48 subjects (33 controls and 15 patients) emigrated during the study period.

### Methods

From the Swedish NPR, information regarding age, sex, comorbidities and treating hospital were collected. All main and subsidiary inpatient-diagnoses according to ICD-10-SE were collected for each unique PE patient for the period 1998 to the index admission for PE in 2005. From altogether 30,061 admissions (including the index admission), ICD-10-SE codes and dates of admissions were extracted and diagnose groups (e.g. malignancies, ischemic heart disease, stroke etc.) were constructed. For details on diagnose groups and ICD-10-SE codes, see Additional file 1: Table S1. We used the 2007 edition of *Nomenclature of Territorial Units for Statistics (NUTS),* created by the European Union for statistical purpose, where Sweden is divided in regions according to county. For detail on Swedish regions and counties, see Additional file 2: Figure S1.

### Statistics

The national and regional (age-adjusted) incidence rates of acute PE are presented with 95% confidence intervals (CI). Differences based on regional rates and their CIs vs. the national rate are reported. For the survival analysis, date of death, censoring of emigration or being alive 23th of April 2010, whatever occurred first, is reported. The actuarial and Kaplan Mayer’s methods were used. Overall comparisons between groups were tested with log ranks tests. To evaluate the impact of follow-up, the analysis was repeated after 3 months and after 1, 2, and 3 years by analysing the survival of all remaining survivors at each time point. Furthermore, survival within the PE group was analysed stratified for the presence of malignant disease.

The six most common comorbidities in each age group based on age quartiles and stratified for sex are presented as percentage with 95% CIs. The statistical software SPSS 21.0 (IBM, Armonk, NY, USA) was used.

## Results

In total, 5793 patients were diagnosed with a main or subsidiary diagnosis of an acute PE in Sweden during 2005, which corresponds to a nation-wide incidence of 0.6/1000/year.

Sixty-nine percent (*n* = 4003) had acute PE as the main diagnosis.

The age ranged from 1 to 103 years and the mean age was 70.4 years (±15.1 SD). In total, 2788 (48%) were males with a mean age of 72.1 years (±15.3 SD) and 3003 (52%) were females with a mean age of 68.7 years (±14.6 SD). Only 12 patients (0.2%) were younger than 18 years the day of the PE, whereof 10 were over 15 years of age.

**Fig. 1:**
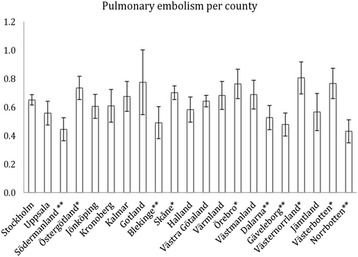
Age-adjusted distribution of PE incidence with 95%CI, divided by county. * = Incidence below the national average. ** = Incidence above the national average

The incidence rates stratified for counties are shown in Fig. 1. The counties of Västernorrland, Västerbotten, Östergötland, Skåne and Örebro had rates above the national rate whereas Dalarna, Blekinge, Gävleborg, Södermanland and Norrbotten had rates below. No clear north-south gradient was seen

The most frequent subsidiary diagnoses registered from 1998 to the index event in 2005 were cardiac diseases, vascular diseases, injuries, malignancies, infectious diseases and gastrointestinal diseases. For definition of groups, see Additional file 1: Table S1 and Tables 1 and 2. Cardiac diseases were the most frequent diagnoses in both men and women and in almost all age groups, and the five most frequent sub-groups were arterial hypertension, arrhythmias, ischaemic heart disease, heart failure, and valvular disease. Within the group vascular diseases, the prevalence of cerebrovascular diseases increased with age in both men and women. The prevalence of inflammatory bowel disease (IBD) was surprisingly low within the group of gastrointestinal diseases. Malignancies and injuries were not further subdivided in this analysis.

**Table 1:** The six most frequent comorbidities registered stratified by age men

	Age quartiles (years)			
Disease groups	−60.9	61–71.9	72–80.9	81-
Cardiac diseases	24 (20–27)	48 (44–52)	60 (57–64)	73 (70–76)
Hypertensive diseases	11 (9–13)	25 (21–28)	30 (26–33)	29 (25–32)
Ischemic heart diseases	6 (4–7)	17 (14–19)	27 (24–31)	36 (33–40)
Arrhythmias	7 (5–9)	17 (14–20)	23 (20–26)	34 (30–37)
Heart failure	3 (2–4)	10 (8–12)	21 (18–24)	35 (32–39)
Valvular diseases (nonrheumatic)	1 (0–2)	2 (1–3)	3 (2–4)	5 (4–7)
Other cardiac diseases	4 (3–6)	4 (3–6)	4 (2–5)	5 (3–7)
Vascular diseases	28 (25–31)	33 (30–37)	34 (31–38)	38 (34–41)
Cerebrovascular diseases	4 (3–6)	10 (8–13)	14 (12–17)	20 (17–23)
Other arterial diseases	3 (2–4)	4 (3–6)	5 (4–7)	7 (5–9)
Other venous diseases	22 (19–25)	21 (18–24)	16 (13–19)	15 (12–18)
Other vascular diseases	0 (0–1)	1 (0–2)	3 (1–4)	4 (3–6)
Gastrointestinal diseases	18 (15–21)	21 (18–24)	24 (21–27)	30 (27–34)
Inflammatory bowel diseases	3 (2–4)	2 (1–3)	2 (1–3)	2 (1–3)
Infectious diseases	23 (20–26)	30 (26–33)	34 (30–37)	43 (39–46)
Injuries	18 (16–21)	20 (17–23)	22 (19–25)	31 (28–34)
Malignancies	17 (14–19)	30 (26–33)	28 (25–32)	22 (19–25)

Numbers shown are percentages with (95% confidence intervals). The ICD-10-SW codes used for defining the disease groups were following: hypertensive diseases 110–115, Ischemic heart diseases 120–125, heart failure 150, valvular diseases 133–139, and other cardiac diseases 105–108, 127–128, 130–131, 140–143, 151. Vascular diseases included cerebrovascular diseases 160–169, other arterial diseases 170–179, other venous diseases 180–189 and other vascular diseases 195–199. Gastrointestinal diseases were defined by K00-K93 whereof inflammatory bowel diseases by K50-K52. Infectious diseases included A00-B99 and J00-J22, injuries 500–599 and T00-T99, and malignanciesC01-C97

**Table 2:** The six most frequent comorbidities registered stratified by age women

	Age quartiles (years)			
Disease groups	−60.9	61–71.9	72–80.9	81-
Cardiac diseases	22 (19–25)	52 (48–55)	65 (62–68)	71 (68–74)
Hypertensive diseases	11 (9–13)	30 (26–33)	38 (34–41)	33 (29–36)
Ischemic heart diseases	6 (4–7)	19 (16–22)	27 (24–31)	34 (30–37)
Arrhythmias	5 (3–6)	16 (13–18)	24 (21–27)	34 (31–38)
Heart failure	2 (1–3)	12 (9–14)	23 (20–26)	36 (33–39)
Valvular diseases (nonrheumatic)	1 (0–2)	2 (1–4)	3 (2–4)	5 (3–7)
Other cardiac diseases	4 (3–5)	4 (2–5)	4 (2–5)	3 (2–4)
Vascular diseases	22 (19–25)	27 (23–30)	32 (29–35)	33 (30–36)
Cerebrovascular diseases	4 (3–5)	10 (8–13)	15 (13–18)	17 (15–20)
Other arterial diseases	3 (2–4)	4 (3–6)	5 (4–7)	7 (5–9)
Other venous diseases	16 (14–19)	14 (11–16)	14 (12–17)	13 (11–16)
Other vascular diseases	1 (0–1)	1 (0–2)	3 (2–4)	3 (2–4)
Gastrointestinal diseases	19 (16–22)	23 (20–26)	27 (24–30)	31 (27–34)
Inflammatory bowel diseases	3 (2–5)	2 (1–3)	2 (1–3)	2 (1–3)
Infectious diseases	24 (21–27)	28 (25–32)	35 (37–39)	39 (35–42)
Injuries	19 (16–22)	23 (20–27)	27 (24–30)	42 (38–45)
Malignancies	27 (24–30)	28 (25–31)	71 (68–74)	15 (12–17)

Numbers shown are percentages with (95% confidence intervals). The ICD-10-SW codes used for defining the disease groups were following: hypertensive diseases 110–115, Ischemic heart diseases 120–125, heart failure 150, valvular diseases 133–139, and other cardiac diseases 105–108, 127–128, 130–131, 140–143, 151. Vascular diseases included cerebrovascular diseases 160–169, other arterial diseases 170–179, other venous diseases 180–189 and other vascular diseases 195–199. Gastrointestinal diseases were defined by K00-K93 whereof inflammatory bowel diseases by K50-K52. Infectious diseases included A00-B99 and J00-J22, injuries 500–599 and T00-T99, and malignanciesC01-C97

Notably, 1141 (20%) patients did not have any registered episode of in-patient care between 1998 and 2005.

**Fig. 2:**
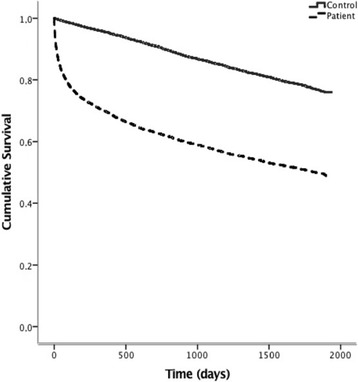
Kaplan-Meier curve demonstrating survival after an acute PE compared to an age-sex- and location matched control group. Log-Rank Test: *p* = <0.001

**Fig. 3:**
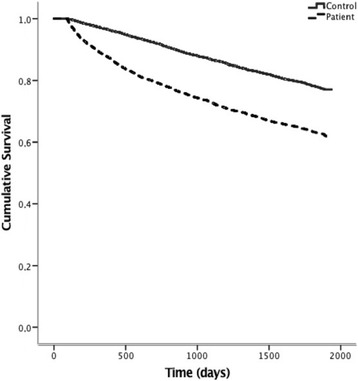
Kaplan-Meier curve demonstrating survival starting 90 days after an acute PE, compared to an age-sex- and location matched control group. Log-Rank Test: *p* = <0.001

**Fig. 4:**
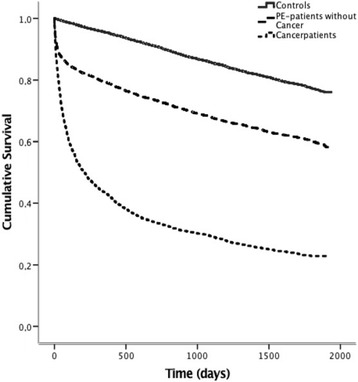
Kaplan-Meier curve demonstrating survival after an acute PE and with no previous history of malignancy, compared to age-sex- and location matched control group. Log-Rank Test: *p* = <0.001

Until 23th April 2010, 2841 (50%) patients died, whereas 1266 (22%) of the matched controls died, thus demonstrating a more than doubled mortality rate in patients with a recent PE (*p* < 0.001), *see* Fig. 2. During the first 3 months, 1204 (21%) patients died and 77 (1.3%) control subjects died. After exclusion of these early deaths, 1637 of 4587 (36%) patients died and 1189 of 4712 (25%) controls died (*p* < 0.001) from three months and onwards, *see* Fig. 3. This procedure was repeated after 1, 2 and 3 years and each analysis showed higher mortality rate in PE patients vs. controls (all *p* < 0.001). The excess mortality amongst PE patients remained after exclusion of all patents with a subsidiary diagnosis of any malignant disease (*p* < 0.001), *see* Fig. 4.

## Discussion

We found a nation-wide incidence of PE of 0.6 per thousand person years in Sweden, which is in line with previous reports [9–12, 21, 22, 29]. Previous estimates have, like ours, been based on retrospective data from hospital records and varying estimates could be explained by varying inclusion criteria. There are challenges to estimate the true the incidence of PE, as the estimated number of unreported cases is likely to be substantial. We do suspect that information from nursing homes and long term care facilities are not found in central registers and our study, like many other, shows that the incidence of PE is strongly correlated to increasing age [9, 10, 22, 29]. Furthermore, the symptoms related to an acute PE are unspecific and mimic most cardiovascular and pulmonary diseases, and the autopsy rate in many countries, like in Sweden, is steadily declining. It should also be emphasised that the events in this study are not validated vs. hospital records.

To our knowledge, the regional differences in incidence of PE in Sweden have not been demonstrated previously. There was no clear-cut difference between the northern part vs. the southern part, neither was there any clear difference between counties with low vs. high populations density and the observed differences were thus probably not due to access to medical care. However, the differences could illustrate local traditions and merits future studies. The play of chance is of course also a possibility.

The mean age at baseline for the Swedish PE population in 2005 was 70 years emphasising that PE is a common disease in elderly persons and will probably increase with increasing longevity. Females had a higher incidence rate than males and lower median age at baseline. Oral contraceptives, pregnancy, and the post-partum period are risk factor for venous thromboembolism, which may contribute to the lower median age of pulmonary embolism amongst woman [30–33].

Approximately 80% of all PE patients had had at least one episode of in-patient care during the preceding 8 years before the index event and cardiovascular diseases were by far the most common comorbidity registered. Although there are proposed links between arterial cardiac disease and PE [3, 6, 7], this study cannot answer whether the prevalence of IHD is higher in the PE population or not, as we do not have comorbidities registered in the control population. Within the group vascular diseases, venous diseases were the most frequent which is expected as deep venous thrombosis (DVT) and PE are manifestations of the same disease and more than 50% of DVT patients have a simultaneous PE at presentation [34]. Notably, the frequency of DVT is probably underestimated as these patients are diagnosed and treated as out-patients and are not captured by the in-patient register. Registered gastrointestinal diseases were common in patients with a future PE and surprisingly, inflammatory bowel disease (IBD) was not commonly registered. IBD has previously been linked to PE [17–19], but may in our study be underreported since most of these patients are treated as outpatients. We conclude that 20% of the patients did not have any registered episodes of in-hospital care before the index event of PE. Our interpretation is that one fifth of the PE population is healthy before the index event and are thus not eligible for prophylactic measures. However, we cannot exclude that these patients had concomitant diseases only requiring outpatient contacts with the health system. There were no major differences in registered comorbidities between men and women.

The short-term (3 months) and long-time survival rates in this Swedish national cohort of PE patients corresponds well with previous reports [3, 20, 22–26]. We show that PE patients have reduced survival compared to the background population even after excluding early deaths and malignancies. This highlights that the PE population needs careful follow-up. Notably, previous studies have reported inconclusive results regarding the survival of PE patients without any known malignancy [22, 27].

The strengths of this report are the size of this national wide cohort and the completeness as no patients were excluded due to age and comorbidities. However, there are some limitations to be noted. The in-hospital diagnoses of PE were not validated and we have no data on patients with PE treated as out-patients. And the size of the unrecognised PE population as discussed above is not known. The control group was well matched, but unfortunately we do not have data on comorbidities in the control population. Subsequent morbidity and causes of death after the index PE (e.g. diagnosis of a malignancy shortly after the PE) merit further analyses.

## Conclusion

In summary, the incidence of acute PE in Sweden was estimated to be 0.6/1000/year and it is increasing with age. There where no major regional differences in incidence rates and no clear north-south gradient. The six most common comorbidities were cardiac-, vascular-, gastrointestinal-, and infectious diseases, injuries and malignancies, with no major differences between males and females. There was a clear increase in mortality in the PE population compared to a matched control-group and the difference remained after exclusion of deaths during the first three months, and after exclusion of patients with known malignancy. This highlights the importance of a proper structural follow-up after an episode of acute PE, a diagnosis still associated with a poor prognosis.

## Additional files

Additional file 1: Table S1. Diagnosis groups based on ICD-10-SE. (DOCX 25 kb)

Additional file 2: Figure S1. Sweden divided by regions according to NUTS. (JPG 67970 kb)

## Abbreviations

CI: Confidence Interval
CTEPH: Chronic Thromboembolic Pulmonary Hypertension
CVD: Cardiovascular Disease
DVT: Deep Venous Thrombosis
HF: Heart Failure
IBD: Inflammatory Bowel Disease
ICD: International Classification of Diseases
IHD: Ischemic Heart Disease
NPR: National Patient Registry
NUTS: Nomenclature of Territorial Units for Statistics
PE: Pulmonary Embolism
VTE: Venous Thromboembolism
