# Supplementary material for: Incidence of acute pulmonary embolism, related comorbidities and survival; analysis of a Swedish national cohort
Source: BMC Cardiovasc Disord. 2017 Jun 14;17:155. doi: 10.1186/s12872-017-0587-1 (PMC5471722; doi:10.1186/s12872-017-0587-1)
Supplement: Supplementary file 1 — Diagnosis groups based on ICD-10-SE. (DOCX 25 kb) [file 12872_2017_587_MOESM1_ESM.docx]

Pulmonary Embolism and Comorbidities divided in to groups based on ICD-10-SE

Pulmonary embolism I26

Infectious diseases A00-B99, J00-J22

Malignacy C01-C97

Benign neoplasms including cancer in situ D01-D48

Hematological diseases D50-D89

Endocrince, nutritional and metabolic diseases E00-E90

Psychiatric diseases F00-F99

Neurological diseases G00-G99

Diseases of the eye, adnexa, ear and mastoid process H00-H93

Respiratory diseases J30-J99

Gastrointestinal diseases K00-K93

Inflammatory bowel disease (IBD) K50-K52

Dermatological diseases L01-L98

Muscoloskeletal system and connective tissue diseases M00-M99

Genitourinary diseases N00-N99

Injuries S00-S99, T00-T99

Cardiac diseases I00-I25, I27-I52

Hypertensive diseases I10-I15

Ischemic heart disease (IHD) I20-I25

Heart failure (HF) I50

Arrhythmias and conductions disorders I44-I49

Valvular disease (nonrheumatic) I33-I39

Other cardiac diseases I05-I08, I27-I28, I30-I32, I40-I43, I51

Vascular diseases I60-I99

Cerebrovascular disease I60-I69

Other arterial diseases I70-I79

Other venous diseases I80-I89

Other vascular diseases I95-I99

Gynecological, obstetric and perinatal diseases O00-O99, P20-P55

Congenital malformations Q05-Q98
