# Supplementary figures and images for: Incidence of acute pulmonary embolism, related comorbidities and survival; analysis of a Swedish national cohort
Source: BMC Cardiovasc Disord. 2017 Jun 14;17:155. doi: 10.1186/s12872-017-0587-1 (PMC5471722; doi:10.1186/s12872-017-0587-1)

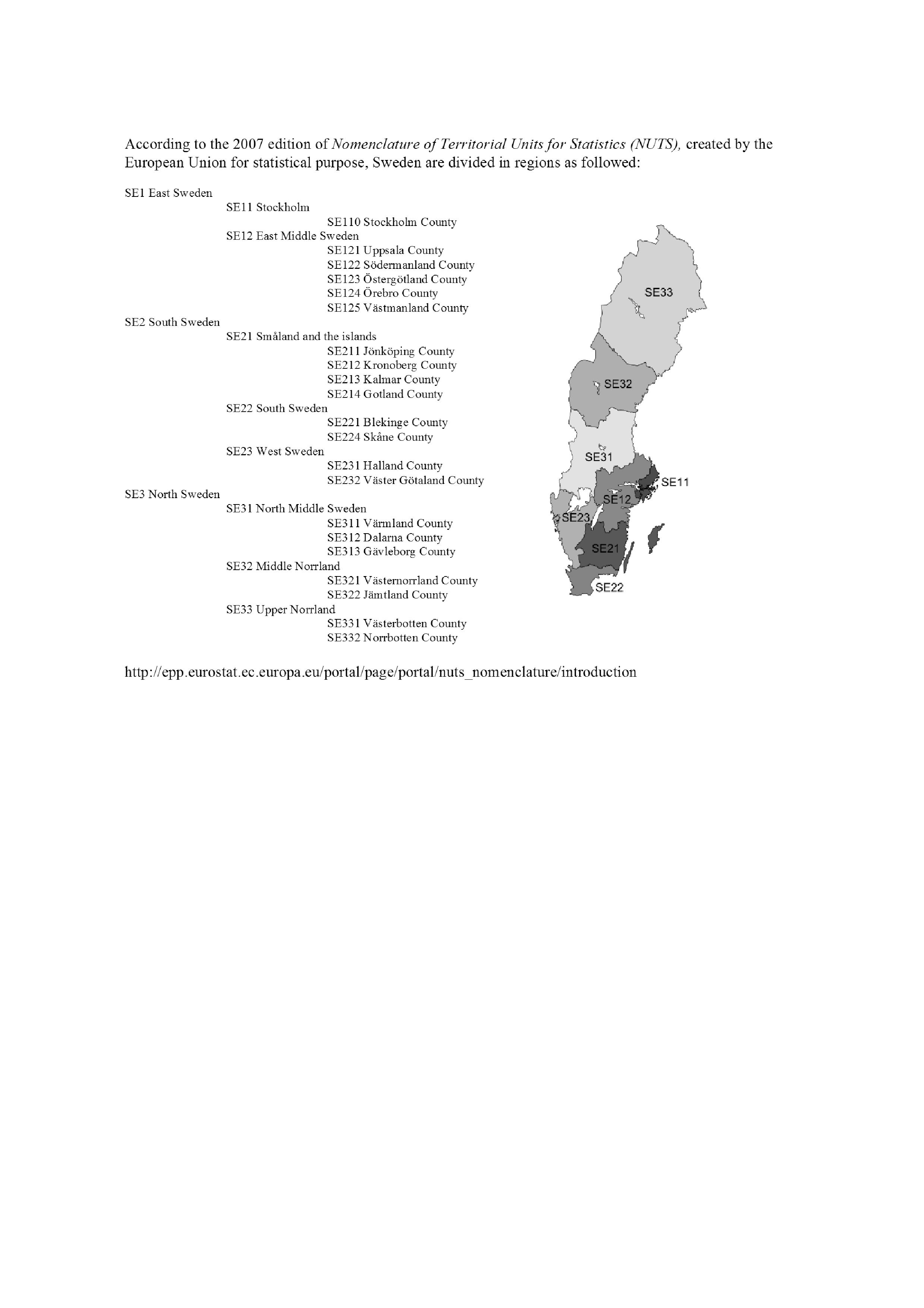

Supplement: Supplementary file 2 — Sweden divided by regions according to NUTS. (JPG 67970 kb) [file 12872_2017_587_MOESM2_ESM.jpg]
